# Cerebral Small Vessel Disease in Immune‐Mediated Thrombotic Thrombocytopenic Purpura Patients During the Acute Phase and Disease Remission

**DOI:** 10.1002/ajh.70506

**Published:** 2026-09-22

**Authors:** Addolorata Truma, Francesco Maria Lo Russo, Giorgio Conte, Ilaria Mancini, Andrea Artoni, Juri Alessandro Giannotta, Barbara Ferrari, Pasquale Agosti, Maria Abbattista, Matteo Gagliardi, Eleonora Piccin, Marco Stroppi, Fabio Maria Triulzi, Flora Peyvandi

**Affiliations:** ^1^ Fondazione IRCCS Ca' Granda Ospedale Maggiore Policlinico, Angelo Bianchi Bonomi Hemophilia and Thrombosis Center Milan Italy; ^2^ Neuroradiology Unit Fondazione IRCCS Ca' Granda Ospedale Maggiore Policlinico Milan Italy; ^3^ Department of Pathophysiology and Transplantation Università Degli Studi di Milano Milan Italy; ^4^ Medical Physics Unit Fondazione IRCCS Ca' Granda Ospedale Maggiore Policlinico Milan Italy

## Abstract

Immune‐mediated thrombotic thrombocytopenic purpura (iTTP) is a life‐threatening thrombotic microangiopathy characterized by acute neurological manifestations and long‐term sequelae related to microvascular brain injury. Cerebral small vessel disease (cSVD), detectable on MRI through lacunes, microbleeds, white matter hyperintensities, and enlarged perivascular spaces, is a risk factor for stroke and cognitive decline, but its prevalence and evolution in iTTP are unknown. We conducted a prospective study of consecutive patients with acute iTTP and age‐matched controls to assess the prevalence and one‐year progression of cSVD. Brain MRI was performed at enrollment and repeated after 12–18 months in clinical remission. cSVD burden was quantified using a standardized 0–4 score based on four MRI markers. Twenty‐five patients (median age 56 years; 60% women) were enrolled. Acute focal ischemic lesions were detected in 13 (52%). cSVD was present in 48% of patients versus 27% of controls, while moderate burden (score ≥ 2) occurred in 24% versus none. Ordinal logistic regression showed an association between iTTP and higher cSVD scores (OR 3.19, 95% CI 1.00–10.14), independent of traditional cardiovascular risk factors. Follow‐up MRI was available in 22 patients (88%). In paired analyses, cSVD prevalence increased from 50% to 64%, moderate‐to‐high burden from 27% to 41%, and six patients (27%) showed score progression. At 1 year, patients had higher odds of greater cSVD burden than controls (age‐ and sex‐adjusted ordinal OR 7.00, 95% CI 1.65–29.74). These findings indicate that cSVD is common during acute iTTP and progresses during the first year despite hematological remission.

## 1 Introduction

Thrombotic thrombocytopenic purpura (TTP) is a rare, life‐threatening thrombotic microangiopathy, with a reported annual incidence of 2–6 cases per million people [1]. Acquired immune‐mediated TTP (iTTP) is caused by severe acquired deficiency of the von Willebrand factor (VWF) cleaving protease, a disintegrin and metalloproteinase with a thrombospondin Type 1 motif, member 13 (ADAMTS13). Severe ADAMTS13 deficiency leads to the accumulation of ultra‐large VWF multimers that, under shear stress, bind platelets to form platelet‐rich thrombi that occlude arterioles and capillaries, causing tissue ischemia [2, 3]. iTTP is characterized by multi‐organ involvement, with a wide spectrum of clinical manifestations. Neurological impairment due to microthrombotic ischemia is reported to be present in approximately 60% of cases and ranges from headache to focal neurological deficits, ischemic stroke (IS), seizures, or coma [4, 5]. Moreover, long‐term survivors of iTTP exhibit reduced survival compared with age‐ and sex‐matched controls, driven by higher rates of cardiovascular comorbidities and major adverse cardiovascular events (MACE), as well as persistent neurological sequelae including cognitive impairment, fatigue, and mood disorders, frequently associated with magnetic resonance imaging (MRI)‐detectable brain alterations [6, 7, 8, 9, 10, 11, 12].

Cerebral small vessel disease (cSVD) refers to a group of pathologies affecting the brain small perforating arterioles, capillaries, and venules. It is common with aging and underlies approximately 25% of ischemic strokes, the majority of spontaneous intracerebral hemorrhages, and is a leading vascular cause of cognitive impairment and dementia [13, 14]. Major risk factors for cSVD include hypertension, aging, and other vascular comorbidities. The most common sporadic forms of cSVD are arteriolosclerosis, due to chronic hypertension and aging, and cerebral amyloid angiopathy, from amyloid‐β deposition in vessel walls. Both cause vessel wall thickening, fragility, and endothelial dysfunction, with blood–brain barrier disruption driving chronic hypoperfusion, inflammation, and microvascular damage [15].

CSVD is diagnosed primarily by its characteristic MRI markers. The STandards for ReportIng Vascular changes on nEuroimaging (STRIVE) criteria describe the classic features: white matter hyperintensities (WMH), lacunes, cerebral microbleeds (CMBs), and enlarged perivascular spaces (EPVS) [16]. The overall cSVD burden can be quantified on MRI using the total cSVD score, which assigns one point for the presence of each predefined imaging marker, thereby providing a composite measure of cumulative microvascular injury. The total cSVD score thus ranges from 0 (no MRI signs of cSVD) up to 4 (all four lesion types present). Large population‐based studies have shown that even a single‐point increase in total cSVD score independently increases the risk of future stroke (HR 1.54), dementia (HR 1.25), and all‐cause mortality (HR 1.15) [17].

The present study aimed to determine the prevalence of cSVD in patients during the acute episode of iTTP, and to assess its radiological progression on follow‐up MRI after 1 year.

## 2 Methods

### 2.1 Study Design and Patients

We performed a single‐center prospective cohort study enrolling consecutive adult patients hospitalized with a diagnosis of acute iTTP between January 2023 and July 2025. All patients underwent brain MRI during hospitalization for the acute event. MRI was repeated during disease remission in patients with a follow‐up of at least 12 months. Exclusion criteria included pre‐existing neurological disorders, major cerebrovascular disease, and MRI contraindications. Age‐frequency matched controls consisted of healthy volunteers or individuals undergoing MRI for otological indications without known neurological disease.

The diagnosis of iTTP episode was defined by the presence of both consumptive thrombocytopenia and microangiopathic hemolytic anemia, in the absence of alternative causes, confirmed by the severe plasma deficiency of ADAMTS13 activity (< 10 IU/dL or < 10% of normal) and evidence of anti‐ADAMTS13 antibodies or normalization of ADAMTS13 activity during follow‐up [18]. ADAMTS13 activity and anti‐ADAMTS13 antibodies were monitored from clinical remission throughout follow‐up according to national and international guidelines [19, 20, 21]. For all patients, ADAMTS13 testing was performed at our center. ADAMTS13 activity was measured by HemosIL AcuStar ADAMTS13 Activity assay (Werfen, Bedford, Massachusetts, United States), as previously reported [22]. The presence of anti‐ADAMTS13 IgG was assessed by the Technozym ADAMTS13 Inhibitor ELISA kit (Technoclone, Vienna, Austria), following the manufacturer's instructions. According to our institutional follow‐up protocol, ADAMTS13 activity was monitored weekly while patients were receiving caplacizumab. After laboratory remission (defined as ADAMTS13 activity > 20 IU/dL) was achieved, ADAMTS13 activity was routinely measured every 3 months during the first year of follow‐up. Remission ADAMTS13 activity metrics were subsequently estimated by linear interpolation between consecutive measurements.

Written informed consent was obtained from all subjects, in accordance with the Helsinki Declaration.

### 2.2 Outcomes and Variables

We recorded age, sex, ethnicity, weight, height, cardiovascular risk factors, TTP‐related variables (first versus relapsing episode, neurological involvement), and neuroimaging data of each patient included in the study. Neurological signs and symptoms, including seizures, coma, focal neurological signs, dizziness, dysarthria/aphasia, confusion, and headache (only if associated with other symptoms), were extracted retrospectively from hospital discharge summaries. The anatomical correspondence between DWI‐positive lesions and focal neurological symptoms at presentation was assessed descriptively based on lesion location and clinical examination. Cardiovascular risk factors (i.e., hypertension, dyslipidemia, Type 2 diabetes, smoking status) as well as a history of cardiovascular disease were also recorded based on documented diagnosis or current daily therapy. Obesity was defined as body‐mass index (BMI) ≥ 30 kg/m^2^.

The primary outcome was the total cSVD score on MRI performed during the acute phase of disease. Secondary outcomes included the total cSVD score on MRI at 12–18 months follow‐up and acute ischemic lesions (diffusion‐weighted imaging DWI‐positive) on acute phase MRI.

### 2.3 Neuroimaging Acquisition and Rating

Brain MRI was performed during hospitalization for the acute event and at 12–18 months of follow‐up on a Philips Achieva 3.0 Tesla MRI scanner using a standardized protocol (Table S1). Acute ischemic lesions were defined as hyperintense areas on DWI with corresponding hypointensity on apparent diffusion coefficient (ADC) maps. DWI‐positive lesions were assessed separately and were not included in the total cSVD score. Only chronic WMH, graded according to the Fazekas scale, were included in the total cSVD score. WMH were defined as patchy or confluent hyperintense areas on T2‐weighted or FLAIR MRI in periventricular or deep white matter, caused by chronic ischemia and loss of myelin. WMH severity was graded visually using the Fazekas scale; a score of ≥ 3 in periventricular areas or ≥ 2 in deep white matter was considered severe (high‐grade) WMH [23]. Lacunes were defined as small cavities (typically 3–15 mm in diameter) in deep gray or white matter, with cerebrospinal fluid–like signal on MRI (T2 hyperintense, T1 hypointense) [24]. CMBs appeared as punctate hypointense foci on T2*‐weighted or susceptibility‐weighted MRI, usually 2–5 mm in size (up to ~10 mm) [25]. EPVS, also known as Virchow–Robin spaces, were identified as fluid‐filled spaces surrounding perforating vessels appearing on T2‐weighted MRI as small (< 3 mm) cerebrospinal fluid–like round or linear spots in basal ganglia, centrum semiovale, or other subcortical areas. A high EPVS burden, defined as > 10 or “Grade 2–4” EPVS in the basal ganglia on a standardized scale, was considered indicative of severe small vessel disease [26]. The total cSVD score was calculated by assigning 1 point for the presence of each of the following: ≥ 1 lacunes, ≥ 1 CMBs, high‐grade WMH, and high‐grade EPVS in the basal ganglia. Multiple lesions of the same type did not increase the score. The total score ranged from 0 (no MRI signs of cSVD) to 4 (all four lesion types present) [16]. For descriptive purposes, the score was categorized as no burden (0), low burden (1), moderate burden (2–3), and high burden (4).

All MRI scans were independently reviewed by two experienced neuroradiologists. Discrepancies were resolved by consensus.

### 2.4 Statistical Analysis

Baseline clinical and neuroimaging features were summarized descriptively. The primary association between iTTP and cSVD was evaluated with binary logistic regression, using cSVD as a dichotomous outcome (total cSVD score ≥ 1 vs. 0), with and without adjustment for the confounding effect of sex and age. As a prespecified exploratory analysis, we also fit a proportional odds (ordinal) logistic regression model, with cSVD score coded as a three‐level ordinal variable (0, 1, ≥ 2) to reflect disease severity. The same binary and ordinal logistic regression models were applied to patients with available one‐year MRI, comparing their follow‐up cSVD burden with that of controls. These models were fitted without adjustment and with adjustment for age at the MRI examination and sex. Given the small sample and sparse cells, proportional‐odds assumptions and model stability were assessed before reporting ordinal regression results. Logistic regression results are presented as odds ratios (OR) with 95% confidence intervals (CI).

Because multivariable models beyond age and sex were at risk of overfitting due to the limited sample size, we explored potential confounding and effect modification by traditional cardiovascular risk factors, as well as by sex and age group, using stratified descriptive tables and stratum‐specific ORs (2 × 2 tables of iTTP vs. control by cSVD present/absent within levels of the modifier) with Fisher's exact tests, where applicable. No formal test of interaction was performed due to limited power.

Hypertension was considered a potential mediator; we therefore estimated the controlled direct effect by restricting to non‐hypertensive participants. This restricted analysis was performed both for acute‐phase and one‐year cSVD outcomes. Additionally, in an exploratory analysis restricted to iTTP cases, we assessed the association between neurological symptoms at acute iTTP presentation and acute ischemic lesions on MRI (DWI‐positive vs. DWI‐negative) using logistic regression, unadjusted and adjusted for age and sex. Finally, to explore the relationship between ADAMTS13 recovery and cSVD evolution, we performed an exploratory intra‐patient analysis in the subgroup of subjects with available follow‐up MRI. For each patient, the percentage of time spent below or above predefined ADAMTS13 activity thresholds (< 20 IU/dL, 20–50 IU/dL and > 50 IU/dL) during clinical remission was calculated by linear interpolation between consecutive measurements. These thresholds were selected to reflect the lack of ADAMTS13 remission (< 20 IU/dL), partial (20–50 IU/dL) and complete (> 50 IU/dL) ADAMTS13 remission [27, 28]. Associations between time‐in‐range metrics and cSVD severity at follow‐up MRI were descriptively evaluated given the limited sample size, without formal statistical inference. Descriptive tables were not subjected to hypothesis‐testing comparisons; inferential analyses are reported as effect estimates with 95% confidence intervals. Fisher's exact *p*‐values were retained only for prespecified stratified analyses with sparse data.

The analyses were carried out using IBM SPSS Statistics version 29 (IBM Corp.).

## 3 Results

The study population comprised 25 patients with acute iTTP and 26 controls (Table 1). The median age of iTTP cases was 56 years (IQR 48–70), the majority were female (60%), and 84% were Caucasian. The median BMI was 26 kg/m^2^ (IQR 24–29). Hypertension was present in 40% of patients, hyperlipidemia in 32%, and only two patients (8%) had Type 2 diabetes mellitus. Most cases (84%) represented a first acute iTTP episode. Neurological manifestation at presentation was observed in 80% of patients, including focal neurological signs, seizures, confusion, or dizziness. All patients were treated with upfront caplacizumab, except for two who started treatment later due to a delayed diagnosis after transfer from another hospital. All achieved clinical and ADAMTS13 remission, with a median time to partial remission (ADAMTS13 activity > 20 IU/mL) of 36 days (IQR 29–48).

**TABLE 1:** Baseline and clinical characteristics of study subjects.

	Acute iTTP (*n* = 25)	Controls (*n* = 26)
Female sex, *n* (%)	15 (60)	20 (77)
Age, years, median (IQR)	56 (48–70)	50 (42–57)
Caucasian ethnicity, *n* (%)	21 (84)	26 (100)
Cardiovascular risk factors		
BMI, kg/m^2^, median (IQR)	26 (24–29)	25 (22–26)
Obesity^a^, *n* (%)	4 (16)	3 (12.5)
Hypertension^b^, *n* (%)	10 (40)	0 (0)
Diabetes mellitus^b^, *n* (%)	2 (8)	1 (4)
Hyperlipidemia^b^, *n* (%)	8 (32)	8 (32)
Smoking^a^, *n* (%)	14 (56)	5 (21)
Positive CVD family history^a^, *n* (%)	4 (16)	13 (54)
TTP‐related variables		
First acute TTP event, *n* (%)	21 (84)	—
Neurological involvement at onset, *n* (%)	20 (80)	—

Abbreviations: BMI, body mass index; CVD, cardiovascular disease; IQR interquartile range; TTP, thrombotic thrombocytopenic purpura.

^a^ Information available in all cases and in 24 of 26 controls.

^b^ Information available in all cases and in 25 of 26 controls.

Brain MRI was performed with a median of 7 days (IQR 4–8) after acute iTTP onset. Acute focal infarcts, defined as DWI‐positive lesions, were detected in 13 of 25 patients (52%). No acute focal infarcts were identified in the control group. Lesions were frequently multifocal, most commonly involving the frontal lobes, followed by parietal and occipital regions; subcortical and deep gray matter structures were occasionally involved (Table 2). Patients with neurological symptoms appeared more likely to have DWI‐positive lesions compared with asymptomatic patients (12/20 [60%] vs. 1/5 [20%]; unadjusted OR 6.0, 95% CI 0.56–63.98; age‐ and sex‐adjusted OR 4.89, 95% CI 0.09–267.75). Among the 13 patients with acute DWI‐positive lesions, 12 (92%) presented with neurological symptoms and, in 10 (77%), the clinical manifestations were anatomically consistent with lesion location. One patient had an apparently clinically silent lesion. Neurological symptoms were also observed in 10 of the 12 patients (83%) without DWI‐positive lesions.

**TABLE 2:** Localization of DWI ischemic lesions in acute iTTP patients.

	Acute iTTP (*n* = 25)
Female sex, *n* (%)	15 (60)
Age, years, median (IQR)	56 (48–70)
Time to MRI, days, median (IQR)	7 (4–8)
DWI‐positive lesion, *n* (%)	13 (52)
Frontal lobe, *n* (%)	9 (69)
Parietal lobe, *n* (%)	4 (31)
Occipital lobe, *n* (%)	2 (15)
Thalamus, *n* (%)	1 (8)
Caudate, *n* (%)	3 (23)
Insula, *n* (%)	1 (8)
Cerebellum, *n* (%)	2 (15)
Splenium of Corpus Callosum, *n* (%)	1 (8)

Abbreviations: DWI, Diffusion‐Weighted Imaging; IQR, interquartile range; MRI, magnetic resonance imaging. Percentages are calculated among DWI‐positive patients (*n* = 13).

Assessment of cSVD in the acute phase showed that 12 of 25 patients had at least one cSVD marker compared with 7 of 26 controls, corresponding to a 48% versus 27% prevalence of cSVD (Table 3, Figure 1). A moderate cSVD burden (total score ≥ 2) was observed in 6 iTTP patients (24%) and in none of the controls. In the acute iTTP cohort, cerebral microbleeds were the most prevalent cSVD feature (32%), followed by high EPVS burden in the basal ganglia and high‐burden WMH (both 20%) and lacunes (8%). cSVD prevalence was similarly increased in both male and female iTTP cases compared with controls (Supplementary Table S2). We observed a more pronounced increase in cSVD scores in iTTP cases compared with controls with advancing age: 56% of iTTP cases aged 50–64 years had a cSVD score of 1 versus 25% of controls, and 75% of those aged > 65 years had a cSVD score ≥ 2 versus none of the controls (Table S3).

**TABLE 3:** Neuroimaging results and total cSVD score in iTTP patients and controls.

	Acute iTTP (*n* = 25)	Controls (*n* = 26)
Female sex, *n* (%)	15 (60)	20 (77)
Age, years, median (IQR)	56 (48–70)	50 (42–57)
Neuroimaging biomarkers		
High‐grade WMH, *n* (%)	5 (20)	1 (4)
Lacunes, *n* (%)	2 (8)	0 (0)
Cerebral microbleeds, *n* (%)	8 (32)	4 (15)
High EPVS burden BG, *n* (%)	5 (20)	2 (8)
Total cSVD score, *n* (%)		
0	13 (52)	19 (73)
1	6 (24)	7 (27)
2	4 (16)	0 (0)
3	2 (8)	0 (0)
4	0 (0)	0 (0)
cSVD burden		
0	13 (52)	19 (73)
Low (1)	6 (24)	7 (27)
Moderate‐high (2–4)	6 (24)	0 (0)

Abbreviations: BG, basal ganglia; CMBs, cerebral microbleeds; cSVD, cerebral small vessel disease; EPVS, enlarged perivascular spaces; IQR, interquartile range; WMH, white matter hyperintensities.

**FIGURE 1:**
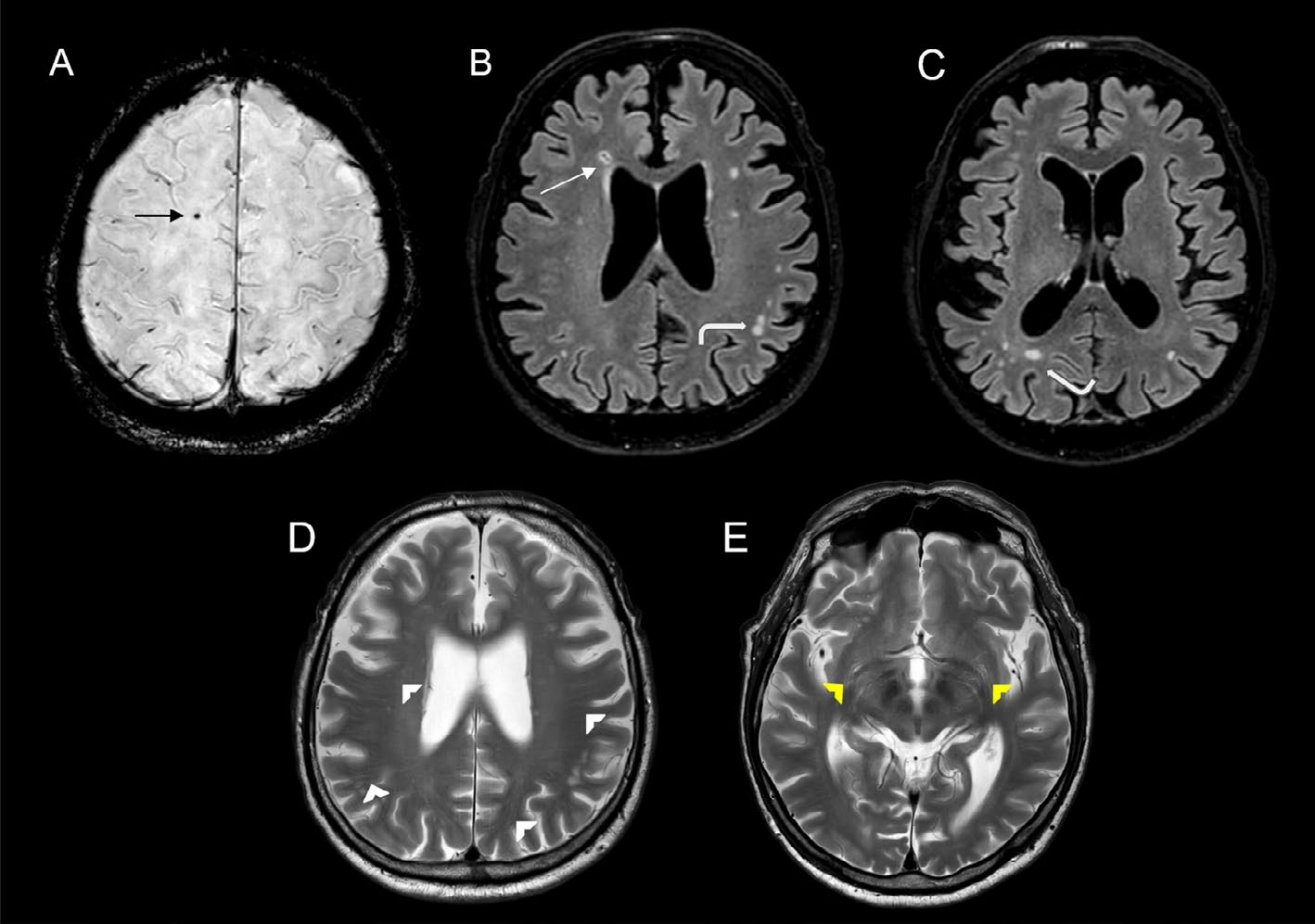
Representative MRI findings of cerebral small vessel disease in an iTTP patient. MRI was performed during the acute phase of disease yielding a total cSVD score of 3. Neuroimaging findings are described as follows. Susceptibility Weighted Imaging (SWI) of a microbleed in the white matter of frontal right lobe (black arrow in Panel A). Axial Fluid Attenuation Inversion Recovery (FLAIR) images show an ischemic lacune in the right frontal periventricular white matter (white arrow, Panel B) and hyperintense lesions both punctate and confluent of subcortical and deep fronto‐parietal white matter consistent with Fazekas grading score 2 (curved arrows, Panel B and C). Axial T2 Turbo Spin Echo (TSE) sequences show some enlarged perivascular spaces in centrum semiovale (white arrowheads, Panel D) and basal ganglia (yellow arrowheads, Panel E). [Color figure can be viewed at wileyonlinelibrary.com]

Binary logistic regression showed a non‐significant trend toward higher odds of cSVD among iTTP patients (unadjusted OR 2.51, 95% CI 0.78–8.07; age‐ and sex‐adjusted OR 2.08, 95% CI 0.56–7.71). Ordinal logistic regression (3‐level cSVD score: 0/1/≥ 2) confirmed the association between iTTP and higher cSVD scores (unadjusted OR 3.19, 95% CI 1.00–10.14; age‐ and sex‐adjusted estimates not reported due to small sample size and sparse data).

In stratified analyses, cSVD prevalence in iTTP patients appeared increased regardless of traditional cardiovascular risk factors (Table S4). Notably, a moderate‐to‐high cSVD burden was present in 5 of 10 iTTP patients with hypertension. After restricting analyses to individuals without hypertension, the association between iTTP and cSVD persisted with attenuated magnitude (age‐ and sex‐adjusted OR 1.67, 95% CI 0.36–7.73).

Twenty‐two of the 25 patients (88%) underwent follow‐up brain MRI at a median of 12.5 months after the acute episode (IQR 12–13 months), whereas three patients were lost to follow‐up. Longitudinal ADAMTS13 data were available for all patients with follow‐up MRI. No DWI‐positive lesions were detected at follow‐up. Compared with the acute MRI, cSVD prevalence increased from 50% to 64%, while moderate‐to‐high cSVD burden (score ≥ 2) increased from 27% to 41% (Table 4). Overall, six of 22 patients (27%) showed progression of the total cSVD score: two progressed from 0 to 1, one from 0 to 2, two from 1 to 2, and one from 2 to 3. Compared with controls, patients at 1 year follow‐up showed a higher prevalence of cSVD (64% vs. 27%). Binary logistic regression yielded an unadjusted OR of 4.75 (95% CI 1.39–16.20) and an age‐ and sex‐adjusted OR of 4.32 (95% CI 0.97–19.19). In the ordinal model, patients at 1 year had higher odds of greater cSVD burden than controls (unadjusted OR 7.00, 95% CI 2.07–23.71; age‐ and sex‐adjusted OR 7.00, 95% CI 1.65–29.74).

**TABLE 4:** Paired cSVD scores in 22 iTTP patients in acute phase and remission.

	Acute	Remission
Female sex, *n* (%)	14 (64)	14 (64)
Age, years, median (IQR)	60 (46–70)	61 (47–71)
Neuroimaging biomarkers		
High‐grade WMH, *n* (%)	5 (23)	5 (23)
Lacunes, *n* (%)	2 (9)	7 (32)
Cerebral microbleeds, *n* (%)	8 (36)	11 (50)
High EPVS burden BG, *n* (%)	4 (18)	5 (23)
Total cSVD score, *n* (%)		
0	11 (50)	8 (36)
1	5 (23)	5 (23)
2	4 (18)	6 (27)
3	2 (9)	3 (14)
4	0 (0)	0 (0)
cSVD burden		
0	11 (50)	8 (36)
Low (1)	5 (23)	5 (23)
Moderate‐high (2–4)	6 (27)	9 (41)

Abbreviations: BG, basal ganglia; CMBs, cerebral microbleeds; cSVD, cerebral small vessel disease; EPVS, enlarged perivascular spaces; IQR, interquartile range; WMH, white matter hyperintensities.

Odds ratios for the presence and increasing burden of cSVD during the acute episode and at 1‐year follow‐up compared with controls are summarized in Figure 2. In the subset of 22 patients with follow‐up MRI, we also calculated the percentage of time spent with ADAMTS13 activity below or above predefined thresholds (< 20 IU/dL, 20–50 IU/dL, > 50 IU/dL) during remission. No association was observed between ADAMTS13 time‐in‐range metrics and cSVD severity (data not shown). Nevertheless, patients with cSVD progression spent a numerically greater proportion of follow‐up time with ADAMTS13 activity below 50 IU/dL than non‐progressors (median 20% [IQR 11.8–61.3] vs. 14% [IQR 6.0–18.5]); given the small number of progressors, this finding should be considered descriptive only.

**FIGURE 2:**
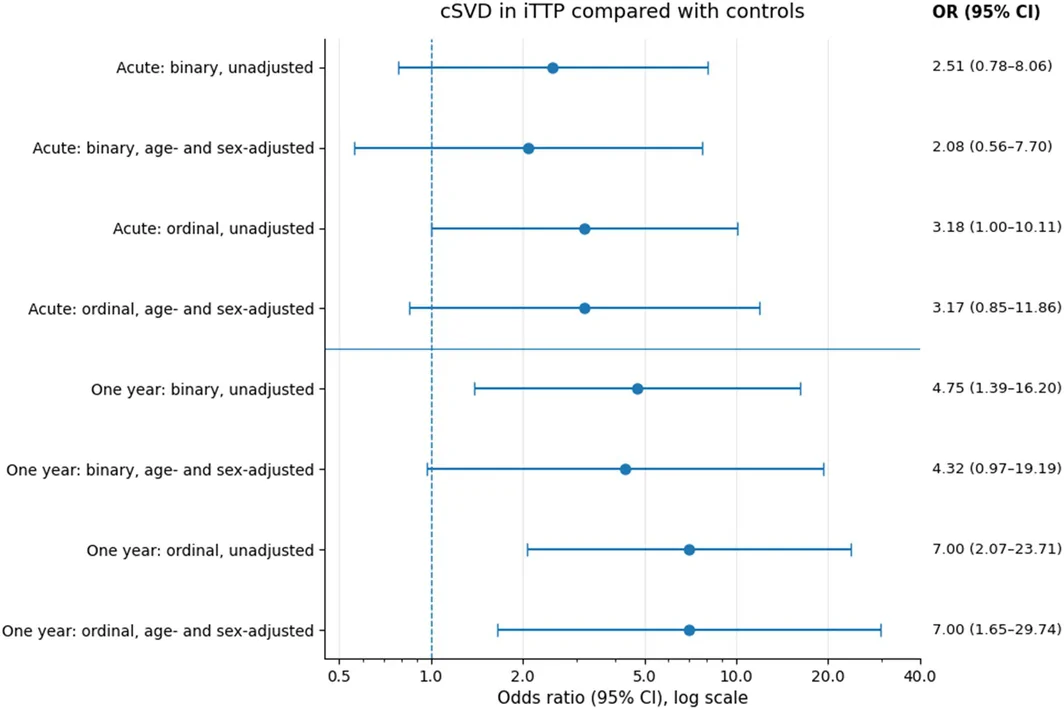
Association of acute and one‐year iTTP with cerebral small vessel disease compared with controls. Forest plot showing unadjusted and age‐ and sex‐adjusted odds ratios with 95% confidence intervals. Binary logistic regression evaluated the presence of cSVD (total cSVD score ≥ 1 vs. 0), whereas ordinal logistic regression evaluated increasing cSVD burden using a three‐level outcome (0, 1, or ≥ 2). The vertical dashed line indicates the null value (OR = 1). CI, confidence interval; cSVD, cerebral small vessel disease; iTTP, immune‐mediated thrombotic thrombocytopenic purpura; OR, odds ratio. [Color figure can be viewed at wileyonlinelibrary.com]

## 4 Discussion

In this prospective study, we evaluated cSVD burden in a well‐characterized cohort of patients with iTTP during the acute episode and at one‐year follow‐up during remission. Acute‐phase MRI detected focal ischemic lesions in 52% of patients, frequently multifocal and involving multiple vascular territories. At baseline, cSVD was present in 48% of patients. Although binary logistic regression did not show a statistically significant association, ordinal analysis suggested greater cSVD burden in patients with iTTP than in controls (OR 3.19, 95% CI 1.00–10.14). Follow‐up MRI demonstrated progression during the first year after the acute episode, with more than one‐quarter of patients showing an increase in total cSVD score.

Although broadly consistent with previous reports, the 52% prevalence of acute ischemic lesions in our cohort was higher than previously reported [5, 8]. This difference may reflect the prospective design of our study and the systematic use of early brain MRI with independent dual neuroradiologist review. In previous retrospective series, neuroimaging was not always performed early or systematically, and brain CT was often used instead of MRI, potentially reducing sensitivity for small or multifocal lesions. Patients presenting with neurological symptoms were more likely to have DWI‐positive ischemic lesions on MRI. Moreover, the clinical presentation was anatomically concordant with lesion location in most patients, supporting the clinical relevance of the MRI findings. However, neurological symptoms were also frequent in patients without DWI‐positive lesions, suggesting that the neurological manifestations of acute iTTP are not solely explained by focal infarction and may also reflect diffuse microvascular injury or other potentially reversible mechanisms.

Previous neuroimaging studies in acute iTTP have mainly focused on acute cerebral complications, such as ischemic stroke and posterior reversible encephalopathy syndrome, whereas the simultaneous assessment of acute ischemic lesions and chronic cSVD has rarely been performed [1, 4, 5]. Our findings indicate that cerebral microvascular injury may already be present at the time of clinical presentation, suggesting that some of these changes may precede the diagnosis of acute iTTP. Comparison with community‐based populations further emphasizes the unexpectedly high cSVD burden observed in our cohort [14, 15, 29]. In the Kashima Scan Study, which included 1349 neurologically healthy adults with a mean age of 58 years, hypertension was present in 33% of participants; 73% had a total cSVD score of 0, 20% a score of 1, and only 7% a score ≥ 2 [30]. The prevalence of moderate‐to‐high cSVD increased with age, reaching 75% among patients older than 65 years, whereas none of the controls in the same age group showed a comparable burden. Moreover, although moderate‐to‐high score was more frequent among hypertensive patients, sensitivity analyses excluding individuals with hypertension yielded similar results, suggesting that traditional vascular risk factors alone are unlikely to fully account for the extent of cerebral microvascular injury observed in iTTP.

Our longitudinal findings complement those of Chaturvedi et al., who reported silent cerebral infarction in approximately half of patients during iTTP remission and demonstrated its association with cognitive impairment [12]. While their study focused specifically on silent cerebral infarction, our study evaluated the broader spectrum of cSVD markers and included both a control group and longitudinal MRI follow‐up. Consistent with these observations, more than one‐quarter of our patients showed progression of the total cSVD score within 1 year, supporting that cerebral microvascular injury continues to evolve after the acute episode. Among patients with progression, the median age was 64 years (IQR 58–71), and four out of six (67%) had hypertension. Progressors also spent a greater proportion of follow‐up time with ADAMTS13 activity below 50 IU/dL than non‐progressors. Although hypothesis‐generating, this finding is biologically plausible, as prolonged exposure to ADAMTS13 activity below 50 IU/dL may contribute to ongoing endothelial injury and cerebral microvascular damage despite clinical remission. However, no association between ADAMTS13 time‐in‐range metrics and the presence or severity of cSVD burden was observed overall. Given the small sample size, these exploratory findings should be interpreted with caution and do not allow conclusions regarding predictors of cSVD progression.

Outside iTTP, higher cSVD burden is associated with stroke, cognitive decline, and mortality [17]. Similarly, iTTP survivors have been reported to experience up to a fivefold higher incidence of ischemic stroke and a six‐fold higher risk of cognitive impairment than the general population during remission [9, 10, 11, 31, 32]. Together with the high prevalence and early progression of cSVD observed in our cohort, these findings raise the possibility that cSVD may represent the biological substrate for this increased stroke risk [9, 10, 11, 31]. One of the premises of the present study was that cSVD may represent a marker of cumulative cerebral microvascular injury and thereby help explain this increased risk of stroke and cognitive impairment. The high prevalence and early progression of cSVD observed in our cohort support this hypothesis, although whether cSVD directly contributes to subsequent ischemic stroke or primarily reflects an underlying diffuse microvascular vulnerability remains to be established. Potential mechanisms may include acute VWF‐mediated microthrombosis, endothelial and blood–brain barrier injury, inflammation, and recurrent subclinical ischemic insults. Strengths of our study include the use of standardized MRI rating scales, independent dual neuroradiologist review, and the inclusion of both acute and follow‐up imaging. Limitations include the small sample size and the single‐center design, which restricted multivariable analyses. Although controls were matched for age and sex, they were not matched for traditional cardiovascular risk factors, and residual confounding cannot be excluded. The low prevalence of some vascular risk factors also limited multivariable analyses. Our findings support MRI‐based assessment of cSVD as a potential tool for long‐term risk stratification in iTTP. Future prospective studies should determine whether cSVD burden predicts long‐term neurological and cognitive outcomes.

## Author Contributions

Addolorata Truma designed the study, enrolled patients, collected clinical data, performed statistical analysis, interpreted the results and wrote the manuscript; Ilaria Mancini designed the study, performed statistical analysis, interpreted the results and critically reviewed the manuscript; Pasquale Agosti designed the study, interpreted the results and critically reviewed the manuscript; Francesco Maria Lo Russo, Eleonora Piccin reviewed MRI scans, analyzed neuroimaging markers and calculated cSVD scores, interpreted the results and critically reviewed the manuscript; Andrea Artoni, Barbara Ferrari, Juri Giannotta enrolled patients, collected clinical data, interpreted the results and critically reviewed the manuscript; Maria Abbattista critically reviewed the manuscript. Matteo Gagliardi performed ADAMTS13 testing and critically reviewed the manuscript. Marco Stroppi performed post‐processing quantitative analysis of MRI data, interpreted the results and critically reviewed the manuscript; Giorgio Conte, Fabio Maria Triulzi interpreted the results and critically reviewed the manuscript; Flora Peyvandi designed the study, interpreted the results and critically reviewed the manuscript.

## Funding

The study was partially supported by the Italian Ministry of Health—Bando Ricerca Corrente and “Piano Nazionale Complementare Ecosistema Innovativo della Salute—Hub Life Science‐Diagnostica Avanzata (HLS‐DA)”—PNC‐E3‐2022‐23683266—“INNOVA.” The Dept. of Pathophysiology and Transplantation, Università degli Studi di Milano is supported by the Italian Ministry of Education and Research ‐ MUR (“Dipartimenti di Eccellenza” Programme 2023–27).

## Ethics Statement

The study was conducted in accordance with the Declaration of Helsinki and was approved by the Institutional Review Board of Fondazione IRCCS Ca' Granda Ospedale Maggiore Policlinico, Milan, Italy, under the following protocols: “Registro internazionale sui pazienti affetti da porpora trombotica trombocitopenica (TTP): caratterizzazione genotipica e fenotipica” (approval no. 1163, approved June 9, 2005) and “Caratterizzazione genetica e fenotipica delle microangiopatie trombotiche” (approval no. 123, approved January 22, 2014).

## Consent

Written informed consent was obtained from all participants prior to study enrollment.

## Conflicts of Interest

I. Mancini received honoraria for participating as a speaker at educational meetings organized by Werfen and Sanofi; A. Artoni received honoraria for participating as a speaker at educational meetings organized by Sanofi and for participating in Advisory Boards organized by Sanofi and Takeda; Juri A. Giannotta, B. Ferrari, and P. Agosti received honoraria for participating as a speaker at educational meetings organized by Sanofi; F. Peyvandi has received honoraria for lectures, presentations, speakers' bureaus, manuscript writing, or educational events by Sanofi, Takeda, Sobi, BioMarin, and Advisory Boards organized by BioMarin, Roche, Sobi, Sanofi, Pfizer, CSL Behring. The other authors declare no conflicts of interest.

## Supporting information


**Table S1:** Acquisition parameters of magnetic resonance imaging (MRI).
**Table S2:** Sex‐stratified analysis of cSVD in acute iTTP patients and controls. Within each sex, the table reports the distribution of the total cSVD score (0–4) and the cSVD burden categories [none (0), low (1), moderate–high (2–4)], with counts and percentages calculated per each sex‐by‐phenotype stratum. For each sex, the OR of having cSVD (score ≥ 1 vs. 0) comparing iTTP cases with controls is reported with 95% confidence intervals (CI) computed by the Woolf method, with Fisher's exact *p*‐values.
**Table S3:** Age‐stratified analysis of cSVD in acute iTTP patients and controls. Within each age group (< 50, 50–64 and ≥ 65 years of age), the table reports the distribution of the total cSVD score (0–4) and the cSVD burden categories [none (0), low (1), moderate–high (2–4)], with counts and percentages calculated per each age group‐by‐phenotype stratum. For each age group, the OR of having cSVD (score ≥ 1 vs. 0) comparing iTTP cases with controls is reported with 95% confidence intervals (CI) computed by the Woolf method, with Fisher's exact *p*‐values.
**Table S4:** Analysis of cSVD in acute iTTP cases and controls stratified by cardiovascular risk factors. Within each factor, the table reports the distribution of the total cSVD score (0–3, as no subject had a score of 4), with counts and percentages calculated per each factor‐by‐ phenotype stratum (per row). For each stratum, the OR of having cSVD (score ≥ 1 vs. 0) comparing iTTP cases with controls is reported with 95% confidence intervals (CI) computed by the Woolf method, with Fisher's exact *p*‐values.

## Data Availability

The data that support the findings of this study are available from the corresponding author upon reasonable request.
