# Supplementary material for: Cerebral Small Vessel Disease in Immune‐Mediated Thrombotic Thrombocytopenic Purpura Patients During the Acute Phase and Disease Remission
Source: Am J Hematol. 2026 Sep 22;101(12):3001–9. doi: 10.1002/ajh.70506 (PMC13742367; doi:10.1002/ajh.70506)
Supplement: Supplementary file 1 [file AJH-101-3001-s001.docx]

# Cerebral small vessel disease in immune-mediated thrombotic thrombocytopenic purpura patients during the acute phase and disease remission

**Supplementary Information**

- **Supplementary methods**
- **Supplementary Table S1.** Acquisition parameters of magnetic resonance imaging (MRI).
- **Supplementary Table S2.** Sex-stratified analysis of cSVD in acute iTTP patients and controls.
- **Supplementary Table S3.** Age-stratified analysis of cSVD in acute iTTP patients and controls.
- **Supplementary Table S4.** Analysis of cSVD in acute iTTP cases and controls stratified by cardiovascular risk factors.

# Supplementary Table S1. Acquisition parameters of magnetic resonance imaging (MRI).

| **Sequence** | **TE**  **(ms)** | **TR**  **(ms)** | **Flip Angle (°)** | **Matrix** | **Voxel Size (mm³)** |
| --- | --- | --- | --- | --- | --- |
| Volumetric T1-weighted TFE | 3.57 | 7.36 | 8 | 256 × 256 | 1 × 1 × 1 |
| Axial T2 | 80 | 3500 | 90 | 1024 × 1024 | 0.22 × 0.22 × 3.5 |
| Volumetric FLAIR | 320.13 | 4800 | 90 | 480 × 480 | 0.52 × 0.52 × 1 |
| 6-echo SWI | 9.8 (TE1); ΔTE=6.8 | 50 | 20 | 512 × 512 | 0.45 × 0.45 × 2 |

Abbreviations: TE, echo time; TR, repetition time; TFE, turbo field echo; FLAIR, Fluid Attenuated Inversion Recovery; SWI, susceptibility weighted imaging; TE1, first echo time; ΔTE, echo spacing.

**Supplementary Table S2. Sex-stratified analysis of cSVD in acute iTTP patients and controls.** Within each sex, the table reports the distribution of the total cSVD score (0–4) and the cSVD burden categories [none (0), low (1), moderate–high (2–4)], with counts and percentages calculated per each sex-by-phenotype stratum. For each sex, the OR of having cSVD (score ≥1 vs 0) comparing iTTP cases with controls is reported with 95% confidence intervals (CI) computed by the Woolf method, with Fisher's exact p-values.

|  | **Females (n=35)** | | **Males (n=16)** | |
| --- | --- | --- | --- | --- |
| Total cSVD score (0-4), n (%) | Cases (n=15) | Controls (n=20) | Cases (n=10) | Controls (n=6) |
| 0 | 8 (53) | 15 (75) | 5 (50) | 4 (67) |
| 1 | 5 (33) | 5 (25) | 1 (10) | 2 (33) |
| 2 | 2 (13) | 0 (0) | 2 (20) | 0 (0) |
| 3 | 0 (0) | 0 (0) | 2 (20) | 0 (0) |
| 4 | 0 (0) | 0 (0) | 0 (0) | 0 (0) |
| cSVD burden, n (%) |  |  |  |  |
| No (0) | 8 (53) | 15 (75) | 5 (50) | 4 (67) |
| Low (1) | 5 (33) | 5 (25) | 1 (10) | 2 (33) |
| Moderate-high (2-4) | 2 (13) | 0 (0) | 4 (40) | 0 (0) |
| OR of cSVD ≥1 vs 0 (95%CI), p-value | 2.63 (0.63–11.00), 0.282 | | 2.00 (0.24–16.36), 0.633 | |

Abbreviations: CI, confidence interval; cSVD, cerebral small vessel disease; OR, odds ratio.

**Supplementary Table S3. Age-stratified analysis of cSVD in acute iTTP patients and controls.** Within each age group (<50, 50-64 and ≥65 years of age), the table reports the distribution of the total cSVD score (0–4) and the cSVD burden categories [none (0), low (1), moderate–high (2–4)], with counts and percentages calculated per each age group-by-phenotype stratum. For each age group, the OR of having cSVD (score ≥1 vs 0) comparing iTTP cases with controls is reported with 95% confidence intervals (CI) computed by the Woolf method, with Fisher's exact p-values.

|  | **<50 years (n=20)** | | **50-64 years (n=17)** | | **≥65 years (n=14)** | |
| --- | --- | --- | --- | --- | --- | --- |
| Total cSVD score (0-4), n (%) | Cases (n=8) | Controls (n=12) | Cases (n=9) | Controls (n=8) | Cases (n=8) | Controls (n=6) |
| 0 | 7 (88) | 9 (75) | 4 (44) | 6 (75) | 2 (25) | 4 (67) |
| 1 | 1 (12) | 3 (25) | 5 (56) | 2 (25) | 0 (0) | 2 (33) |
| 2 | 0 (0) | 0 (0) | 0 (0) | 0 (0) | 4 (50) | 0 (0) |
| 3 | 0 (0) | 0 (0) | 0 (0) | 0 (0) | 2 (25) | 0 (0) |
| 4 | 0 (0) | 0 (0) | 0 (0) | 0 (0) | 0 (0) | 0 (0) |
| cSVD burden, n (%) |  |  |  |  |  |  |
| No (0) | 7 (88) | 9 (75) | 4 (44) | 6 (75) | 2 (25) | 4 (67) |
| Low (1) | 1 (12) | 3 (25) | 5 (56) | 2 (25) | 0 (0) | 2 (33) |
| Moderate-high (2-4) | 0 (0) | 0 (0) | 0 (0) | 0 (0) | 6 (75) | 0 (0) |
| OR of cSVD ≥1 vs 0 - (95% CI), p-value | 0.43 (0.04–5.06), 0.619 | | 3.75 (0.47–29.75), 0.335 | | 6.00 (0.58–61.84), 0.277 | |

Abbreviations: CI, confidence interval; cSVD, cerebral small vessel disease; OR, odds ratio.

**Supplementary Table S4. Analysis of cSVD in acute iTTP cases and controls stratified by cardiovascular risk factors.** Within each factor, the table reports the distribution of the total cSVD score (0-3, as no subject had a score of 4), with counts and percentages calculated per each factor-by- phenotype stratum (per row). For each stratum, the OR of having cSVD (score ≥1 vs 0) comparing iTTP cases with controls is reported with 95% confidence intervals (CI) computed by the Woolf method, with Fisher's exact p-values.

|  | | **Cases (n=25)** | | | | | **Controls (n=26)** | | | | | **cSVD in cases vs controls**  **(score ≥1 vs 0)** |
| --- | --- | --- | --- | --- | --- | --- | --- | --- | --- | --- | --- | --- |
| **Variable** | **Stratum** | **n** | **0** | **1** | **2** | **3** | **n** | **0** | **1** | **2** | **3** | **OR (95% CI), p-value** |
| **Obesity*** | **Yes** | 4 | 2 (50) | 0 (0) | 0 (0) | 2 (50) | 3 | 3 (100) | 0 (0) | 0 (0) | 0 (0) | na, 0.429 |
|  | **No** | 21 | 11 (52) | 6 (29) | 4 (19) | 0 (0) | 21 | 14 (67) | 7 (33) | 0 (0) | 0 (0) | 1.82 (0.52-6.33), 0.530 |
| **Hypertension†** | **Yes** | 10 | 3 (30) | 2 (20) | 3 (30) | 2 (20) | 0 | 0 (0) | 0 (0) | 0 (0) | 0 (0) | na, na |
|  | **No** | 15 | 10 (67) | 4 (27) | 1 (7) | 0 (0) | 25 | 18 (72) | 7 (28) | 0 (0) | 0 (0) | 1.29 (0.32-5.13), 0.736 |
| **Diabetes mellitus†** | **Yes** | 2 | 1 (50) | 0 (0) | 1 (50) | 0 (0) | 1 | 0 (0) | 1 (100) | 0 (0) | 0 (0) | na, 1.000 |
|  | **No** | 23 | 12 (52) | 6 (26) | 3 (13) | 2 (9) | 24 | 18 (75) | 6 (25) | 0 (0) | 0 (0) | 2.75 (0.80-9.45), 0.135 |
| **Hyperlipidemia†** | **Yes** | 8 | 4 (50) | 2 (20) | 2 (20) | 0 (0) | 8 | 5 (62) | 3 (38) | 0 (0) | 0 (0) | 1.67 (0.23-12.22), 1.000 |
|  | **No** | 17 | 9 (53) | 4 (24) | 2 (12) | 2 (12) | 17 | 13 (76) | 4 (24) | 0 (0) | 0 (0) | 2.89 (0.66-12.57), 0.282 |
| **Smoking*** | **Yes** | 14 | 6 (43) | 4 (29) | 3 (21) | 1 (7) | 5 | 5 (100) | 0 (0) | 0 (0) | 0 (0) | na, 0.045 |
|  | **No** | 11 | 7 (64) | 2 (18) | 1 (9) | 1 (9) | 19 | 12 (63) | 7 (37) | 0 (0) | 0 (0) | 0.98 (0.21-4.58), 1.000 |
| **CVD family history*** | **Yes** | 4 | 1 (25) | 1 (25) | 2 (50) | 0 (0) | 13 | 10 (77) | 3 (23) | 0 (0) | 0 (0) | 10 (0.74-135.33), 0.099 |
|  | **No** | 21 | 12 (57) | 5 (24) | 2 (10) | 2 (10) | 11 | 7 (64) | 4 (36) | 0 (0) | 0 (0) | 1.31 (0.29-5.89), 1.000 |

*Available in all cases and in 24 of 26 controls. †Available in all cases and in 25 of 26 controls.

Abbreviations: CI, confidence interval; cSVD, cerebral small vessel disease; CVD, cardiovascular disease; na, not applicable (i.e., not estimable due to zero cell count or lack of variability within the stratum); OR, odds ratio.
